# An Advanced Pneumatic Compression Therapy System Improves Leg Volume and Fluid, Adipose Tissue Thickness, Symptoms, and Quality of Life and Reduces Risk of Lymphedema in Women with Lipedema

**DOI:** 10.3390/life15050725

**Published:** 2025-04-30

**Authors:** Karen L. Herbst, Carlos Zelaya, Marianne Sommerville, Tatiana Zimmerman, Lindy McHutchison

**Affiliations:** 1 The Roxbury Institute, Tucson, AZ 85715, USA; drherbst@theroxburyinstitute.com; 2 Carolina Vein Center, Durham, NC 27713, USA

**Keywords:** lipedema, lymphedema, bioimpedance spectroscopy, ultrasound, extracellular fluid, intracellular fluid, advanced pneumatic compression device, compression therapy

## Abstract

Lipedema is a painful disease of subcutaneous adipose tissue (SAT) in women. This study determined whether an advanced pneumatic compression device (APCD) improved lipedema SAT depth, swelling, and pain. Women with lipedema started 20–30 mm Hg compression leggings then were randomized to an APCD (Lympha Press Optimal Plus) group for 30 days (treatment; n = 22) or a no APCD (Control; n = 24) group. APCD treatment significantly reduced left leg volume (3D imaging, LymphaTech; *p* < 0.043) and fluid in the left (*p* = 0.0018) and right legs (*p* = 0.0476; SOZO, bioimpedance spectroscopy); controls showed no change. Treatment significantly decreased extracellular fluid (ECF) and intracellular fluid (ICF) in left (*p* = 0.0077; *p* = 0.0060) and right legs (*p* = 0.0476; *p* ≤ 0.025), respectively. Only ECF decreased significantly in the left (*p* < 0.0183) and right legs (*p* = 0.0009) in controls. SAT depth decreased significantly by ultrasound after treatment at the anterior (*p* ≤ 0.0234) and medial thigh (*p* ≤ 0.0052), medial knee (*p* ≤ 0.0002) and posterior calf (*p* ≤ 0.0118) but not in controls. All signs and symptoms of lipedema improved in the treatment group including swelling (*p* = 0.0005) and tenderness (pain) of right (*p* = 0.0003) and left legs (*p* < 0.0001); only swelling improved in controls (*p* = 0.0377). In total, 87.5% of RAND SF-36 quality of life improved after treatment (*p* ≤ 0.0351) compared to 37.5% in controls (*p* ≤ 0.0475). APCDs are effective treatment for lipedema.

## 1. Introduction

Lipedema is a disease of connective tissue in women [1,2]. A main feature of lipedema is increased fibrotic subcutaneous adipose tissue (SAT) deposited in the legs, pelvis, abdomen [3], and/or arms of women that is often painful, feels heavy, and can grow to such an extent that it affects mobility [4]; lipedema is rare in men.

Women with lipedema present in the following stages: Stage 1, smooth skin over fibrotic nodular tissue, Stage 2, dimpling or mattress pattern of the skin over larger fibrotic nodules, and Stage 3, lobules of fibrotic nodular lipedema tissue. The location of fat is described through phenotypes, where Phenotype 1 lipedema affects the hips, pelvis, and buttocks, Phenotype II affects hips to knees, Phenotype III affects hips to ankles, Phenotype IV affects the arms, and Phenotype V, which is rare, affects the lower legs only. Most women present with Phenotypes II and IV or III and IV lipedema. Lipedema tissue is difficult to decrease by diet, exercise, or even bariatric surgery [5,6,7] in part due to the high amounts of tissue fibrosis present.

Lymphedema and swelling are notable co-morbidities with lipedema. The risk of lymphedema increases with stage, as well as body mass index (BMI) [3]. The association of lymphedema with lipedema is likely due to increased endothelial cell permeability in micro-vessels from an inflammatory insult [8,9,10], followed by a higher rate of lymphatic vessel pumping rates even in the tissue of women with early-stage lipedema [11]. Other co-morbidities associated with lipedema include allergies [12], attention-deficit/hyperactivity disorder [13], hypermobile joints [14], and vein disease [15].

Conservative management options for lipedema focus on improving lymphatic flow and controlling edema (swelling) with compression garments and manual lymphatic drainage and reducing pain or discomfort by deep tissue therapy to reduce fibrosis [16,17]. Pneumatic compression device therapy is effective in treating symptoms of lipedema, including swelling, easy bruising, and pain [18,19,20,21,22]. These studies used a holistic approach combining decongestive therapy such as tightly wrapping the legs or manual lymphatic drainage (MLD) therapy with use of an APCD. This new study is undertaken to isolate APCD treatment and understand how it affects lipedema apart from MLD and wrapping.

The goal of this study is to determine if in-home use of an APCD can reduce lipedema tissue depth, tissue volume, and fluid content, and improve quality of life, pain, and other signs and symptoms in women with primarily Stage 2 lipedema without evidence of lymphedema or vein disease.

## 2. Materials and Methods

Patient recruitment: The study was conducted according to the guidelines of the Declaration of Helsinki and approved by the WCG Institutional Review Board (protocol code 1356101; date of approval 29 June 2023). Informed consent was obtained from all subjects involved in the study prior to commencement of study activities. Patients seen at the clinic between 2021 and 2023 who had a diagnosis of lipedema and had expressed interest in research were recruited by phone call. Fifty-eight patients were invited to the clinic and presented for duplex scanning to rule out venous disease; 46 returned for the screening visit and signed the consent form. All 46 women completed the study.

Lipedema criteria: Women enrolled in the study met criteria of being a biologic female, having the upper torso spared of abnormal SAT on physical exam, a mismatch between the amount of SAT on the limbs versus the upper torso, symmetrically affected SAT, and hands and feet without evidence of lymphedema per the criteria of Wold et al. [23].

Inclusion criteria:

1. Ambulatory females, age 18–70 years.
2. Stage 2–3, Type II–III lipedema.
3. Pain score with or without pressure in any lipedema area of 3 or more on an 11-point Likert visual analog scale.
4. Able to maintain a consistent eating plan and exercise regimen for the 60-day study with weight stability (within 4.5 kg or usual weight fluctuation per patient) over three months.
5. Willing to wear compression garments during the study.
6. Agreement to wash off manual therapy of any kind including massage, physical therapy, occupational therapy, instrument-assisted soft tissue therapy, or other deep tissue therapy and decongestive therapy methods (manual lymph drainage, pneumatic compression use, and compression garments other than the ones provided) over 30 days prior to Visit 2.

Exclusion criteria:

1. Inability to understand the purpose of the study and complete consent.
2. Bed bound, preventing assessment of activities of daily living.
3. Contraindications to APCD use:
  (a) Serious arterial insufficiency measured as a monophasic pulse wave using Doppler (Terason uSmart 3300-15L4 uSmart Linear Array Transducer; Burlington, MA, USA) without arterial disease.
  (b) Edema due to decompensated congestive heart failure (CHF), based on history or physical examination.
  (c) Active phlebitis, based on physical examination.
  (d) Active deep vein thrombosis, based on history or physical examination.
  (e) Localized wound infection, based on physical examination.
  (f) Cellulitis, based on history or physical examination.
4. Positive Stemmer sign on the feet.
5. Lymphedema with minimal to no lipedema.
6. eight > 170 kg due to weight restriction on bioimpedance spectroscopy device.
7. Undergoing surgery during the time of the study.
8. Weight loss surgery within the past 18 months.
9. Use of diuretic medication.
10. Participation in other research at the time of the study.
11. Use of immunosuppressant medications including Gleevec, diosmin, methotrexate, corticosteroids, Plaquenil, or other.
12. Medical illness deemed significant by the principal investigator.
13. Waist to hip ratio > 0.85 suggestive of obesity with lipedema.

Women in the study had a history of chronic venous insufficiency (CVI) that had been treated previously, except for two participants in the treatment group who did not have CVI and therefore did not have CVI treatment. All women were confirmed to have a lack of venous reflux (effective treatment) with venous duplex ultrasound.

Power calculation: Our power calculation determined that a sample size of 25 participants per group (treatment and control) would provide a statistical power of at least 0.8. This calculation was based on the expected effect size, significance level, and variance estimates, ensuring an adequate probability of detecting a true effect on volume reduction after use of an APCD. The deviation from the recruitment goal was primarily due to time limitations, which affected the recruitment rate and participant availability, leading to a shortfall in the final enrolled numbers.

Randomization: Randomized using Research Randomizer [24] at Visit 1 (V1) into the APCD treatment or control group.

**Figure 1:**
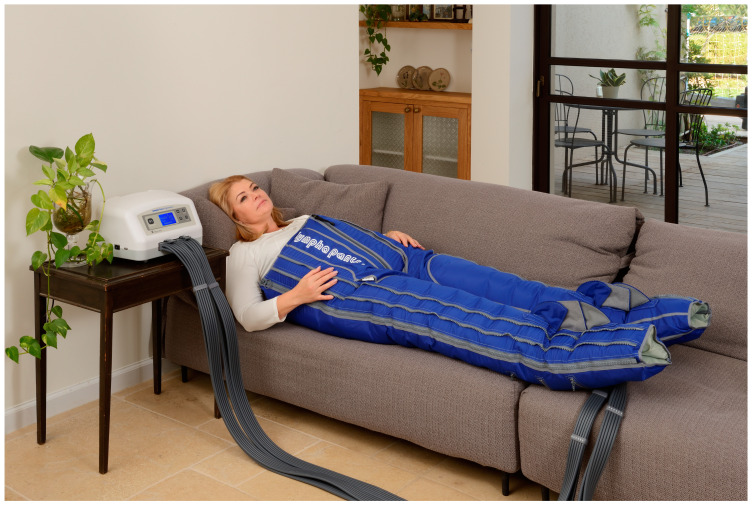
Advanced pneumatic compression (APCD) device studied. The photo is not of a study subject. Used with permission.

Pneumatic compression: The APCD used was Lympha Press^®^ Optimal Plus (LymphaPress, Chadds Ford, PA, USA) (Figure 1). The device is a calibrated gradient compression therapy system allowing a variety of adjustments, including pressure selection and adjustable timing and treatment modes. The compression appliance used (LymphaPants^®^) contains 24 inflatable chambers and treats bilateral legs, hips, buttocks, and abdomen. The pressure used was 45 mmHg, with Sequential or Wave modes chosen according to the patient’s comfort level, with Pretherapy, a proximal decongestive mode, added prior to the main treatment mode. Each APCD includes compliance software that records device usage and settings.

Compression: All women received a standard elastic compression garment (Absolute Support leggings, 20–30 mm Hg; discountsurgical.com) at V1 and wore them daily at home through V3.

Study protocol: The women with lipedema underwent a 30-day washout period starting at Baseline (V1) and were asked not to use any decongestive therapies (manual therapies, pneumatic compression, or compression garments other than the leggings provided for study participants at V1) over the next 30 days; all women were provided compression garments at V1. At Visit 2 (V2), 22 women randomized to the treatment group began the use of the APCD at home for 1–2 h per day during the following 30 days. Women in the treatment group also used the APCD at the clinic in a one-hour session during V2 and Visit 3 (V3), the latter being after 30 days of APCD use; 24 women randomized to the control group did not use the APCD pump at home or during any visit. During the time (60 min) allotted for treatment with the APCD pump, the control subjects reclined in the same position (legs elevated) as used for the treatment group during the APCD pumping sessions and maintained that position for the full 60 min.

Mobility: Women with lipedema often have mobility limitations [25]. Mobility was assessed during V2 and V3 using the following assessments:

- The “Timed Up and Go (TUG)” test [26].
- The lower extremity function scale (LEFS), a questionnaire containing 20 questions about a person’s ability to perform everyday tasks [27].

Caliper measurements: Under the umbilicus, on the anterior thigh and medial thigh at V2 and V3 (Lange, NutriActiva, Minneapolis, MN, USA), as previously measured in women with lipedema [28].

Photos: Photos of the women were taken before and after APCD use or no APCD use at the clinic during V2 And V3.

Leg Volume

Tape measurement: At V2 and V3, with the back of the leg against a measuring grid. Measurements began at the ankle around the malleoli at 10 cm and continued every 10 cm to the upper thigh (70 cm maximum) with, on average, seven measurements total for the lower and upper leg. The volume of the leg was estimated using the formula for the volume of the frustum of a cone [29]. While consistent measurement by the same research staff at each visit is important, due to staff schedules, subjects were measured by different staff at each visit.
3D imaging: Leg volume was measured at V2 and V3 using a 3D imaging system (LymphaTech, Atlanta, GA, USA) [30]. Regarding measurement time, pre was before and post was after treatment (treatment group) or no treatment (control group). The volume of the leg was estimated using the formula for the volume of the frustum of a cone [31].

Bioimpedance spectroscopy (BIS): Fluid/water of the whole body and the legs and the intracellular and extracellular fluid in the legs were assessed at V1–V3 (SOZO^®^ applications, Impedimed, Brisbane, Australia). Bilateral L-Dex^®^ Analysis was used to measure tissue fluid content of the legs and represents the ratio of measured impedance between the arms and legs, with the legs being the at-risk limbs and the arms the lower risk limbs, as compared to an equivalent healthy population, with a normal range from −10 to 10 [32].

RAND short-form 36-item (SF-36) survey (Version 1.0): Administered during V2 and V3 and scored as previously published [33].

Visual analog scales (VAS): During V2 and V3, women filled in an 11-point Likert visual analog scale (range 0–10) to assess the following symptoms in the legs: tenderness, aching, or throbbing, burning or stinging, tiredness or heaviness, swelling or tightness of the legs, difficulty walking, and easy bruising.

Ultrasound assessment of adipose tissue depth: Adipose tissue depth was assessed from the bottom of the skin to the deep fascia during V2 and V3 using a Terason uSmart 3300 ultrasound unit (Burlington, MA, USA). A 15L4 uSmart Linear Array transducer was placed perpendicular to the skin in a transverse plane. Six locations were assessed in areas typically affected by lipedema by the same individual throughout the study: anterior thigh midpoint between the groin and the superior aspect of the patella, lateral thigh measured directly over the head of the femur, medial thigh measured directly down from the midpoint measurement on the anterior thigh, medial knee, fat pad under the knee measured from the inferior patella, and posterior calf at the largest point, with the measurement logged as distance from the popliteal space and the medial malleolus.

Statistics: Data are presented as mean ± standard deviation (SD). The differences between measures within a visit or between two visits for the same group (treatment or control) were evaluated using paired *t*-tests or RM-ANOVA with Tukey’s multiple comparison test for parametric data (between 3 and 4 evaluations). The significance of data was set to *p* < 0.05. Figures show significant differences only. Statistical analyses were performed using GraphPad Prism version 10.0.0 for Windows, GraphPad Software, Boston, MA, USA, www.graphpad.com.

## 3. Results

**Figure 2:**
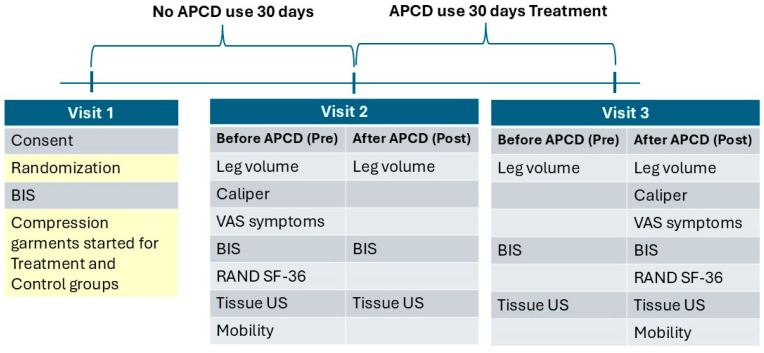
Study layout. Procedures during Visit 1 (Baseline), Visit 2 with Pre (before APCD use) and Post (after APCD use) time points, and Visit 3 with time points pre and post APCD use. The control group followed the same process, without home use of an APCD, and during visits, they reclined for 60 min in the same position as the treatment group without performing APCD therapy. BIS = bioimpedance spectroscopy; US = ultrasound; VAS = visual analog scale.

Starting at Baseline (V1) all women in the study underwent an initial 30-day washout period where they wore 20–30 mm Hg leggings and did not use an APCD (Figure 2). After 30 days, starting at V2, 22 women were randomized to use an APCD at home during the following 30 days in addition to a one-hour session (treatment group) at the start of V2 and V3; the remainder of the women were in the control group (*n* = 24) and did not use an APCD pump at home or during any visit.

### 3.1. Compliance

Twenty of twenty-two women from the treatment group had data that could be downloaded from the APCD compliance software. Sixteen were 100% compliant (daily use), two were 75–89% compliant (five–six days/week treatment), and two were 50–74% compliant.

### 3.2. Demographics

Forty-six women were randomized to the treatment group (APCD for 30 days) or the control group (Table 1). The groups were well matched without significant differences in variables (Table 1). The high numbers of women with anxiety and birth control use were notable.

### 3.3. Quantitative Measures

#### 3.3.1. Leg Volume

##### Tape Measure

There was no significant change in mean tape measure volume from V2 to V3 for the treatment group left (5.8 ± 1.6 to 5.9 ± 1.6 L) or right leg (5.7 ± 1.4 to 5.9 ± 1.6 L), or for the control group left leg (5.5 ± 1.7 L to 5.7 ± 1.8) or right leg (5.6 ± 1.7 L to 5.7 ± 1.5 L).

##### Three-Dimensional Leg Imaging

**Figure 3:**
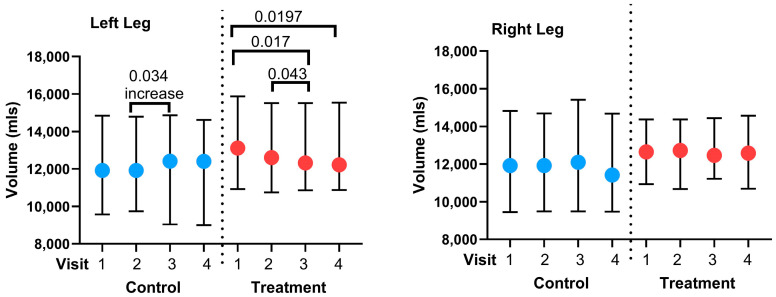
Leg volume measurements before and after advanced pneumatic compression device use in the treatment group or after no intervention in the control group. Volumes (mean ± SD) were assessed as pre and post APCD (red) or no intervention (blue) in Visit 2 (V2) and pre and post APCD in Visit 3 (V3) using 3D imaging).

**Figure 4:**
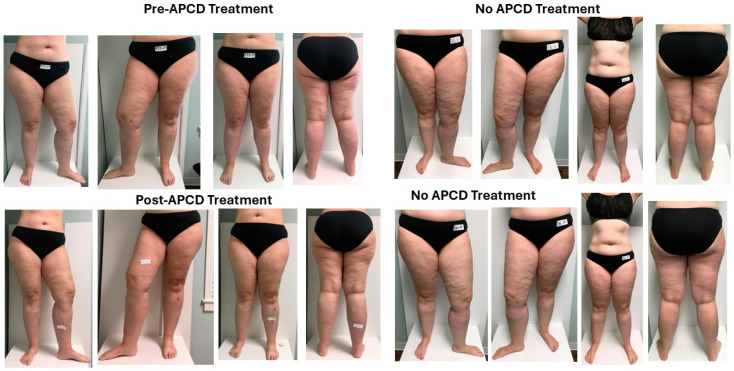
Photos of a women with lipedema before and after treatment with an APCD or no treatment (Control). Top row left four photos: photos prior to treatment for one hour with an APCD during the study visit in clinic. Bottom row left four photos: photos after one-hour treatment with an APCD. Top row right four photos: photos prior to no treatment with an APCD (control). Bottom row right: four photos taken after sitting with legs up for one hour (control).

To determine if there was a change in the volume of the legs after home use of the APCD, we evaluated volume before the 30 days of APCD use at home (pre-V2) and after 30 days of APCD use at home in the treatment group (pre-V3) using paired t-tests. This avoided any effect of the in-clinic use of the pump (post-V2 and post-V3). There was a significant reduction in left leg volume observed by 3D imaging from pre-V2 to pre-V3 (*p* = 0.017; Figure 3), suggesting effective volume reduction by home use of the APCD. There was no significant reduction in volume pre-V2 to pre-V3 for the right leg of the treatment group or for the right and left legs of the control group. Another way to evaluate changes in the volume of the legs when the women were at home for 30 days with or without an APCD is to compare leg volumes starting from the end of V2 (post-V2) to the beginning of V3 (pre-V3). This analysis evaluates the last volume at the time the women left the clinic in V2 to the first volume checked when they returned for V3. There was a significant decrease in left leg volume by 3D imaging for the treatment group, and a significant increase in left leg volume for the control group (Figure 3) despite the fact that the women were wearing compression garments daily during this time. There was no change in the volume of the right legs for either the control group or the treatment group from post-V2 to pre-V3 (Figure 4). We also tested to see if leg volume changed over the course of the entire study from the start of V2 (pre-V2) to the end of the study at V3 (post-V3). There was a significant reduction in volume of the left legs in the treatment group but not in the left legs from the control group using this comparison. There was also not a significant change in volume for the right legs in either the treatment or control group from pre-V2 to post-V3. There were no significant changes in the left or right legs for the treatment group after in-clinic use of the APCD. There were also no significant changes in the right or left legs in the control group after sitting with their legs up in clinic.

##### Weight

There was no significant difference in body weight in the control group between V1 (94.2 ± 23.7 kg), pre-V2 (93.8 ± 23.8 kg), and pre-V3 (94.0 ± 24.5 kg; *p* = 0.05923) or in the treatment group between V1 (104.6 ± 20.9 kg), pre-V2 (105.1 ± 20.9), and pre-V3 (104.8 ± 21 kg; *p* = 0.32).

#### 3.3.2. Bioimpedance Spectroscopy

##### L-Dex Score

**Figure 5:**
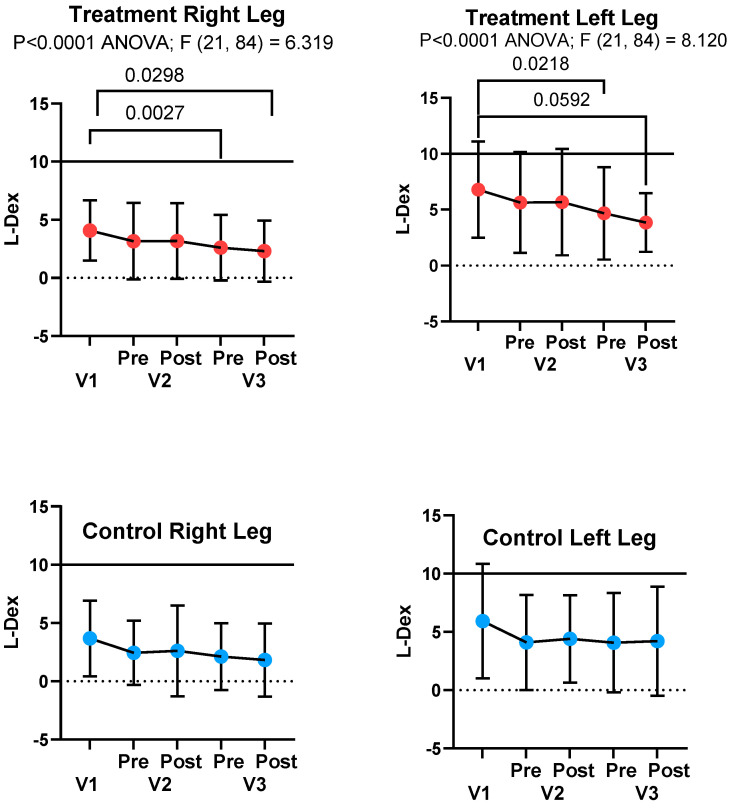
L-Dex measurement (SOZO, Impedimed, Brisbane, Australia) during Baseline Visit 1 (V1), Visit 2 (V2), before 30 days of advanced pneumatic compression device (APCD) use in the treatment group, and Visit 3 (V3), after APCD use (treatment group) or no APCD use (controls). During V2 and V3, L-Dex measures were pre and post either resting (control; blue) or use of the APCD for one hour (treatment; red). Values below the line at an L-Dex score of 10 on the graph are within normal limits.

There was a significant decrease in the potential risk of lymphedema (L-Dex Score; SOZO) in the treatment group for the right and left legs, but not in the control group, between V1 and V3 (Figure 5). L-Dex scores for the right and left legs were significantly different at V1 for the treatment group (4.1 ± 2.6 and 6.8 ± 4.3; *p* < 0.0001) and control group (3.7 ± 3.2 and 5.9 ± 4.9; *p* < 0.0001), respectively.

##### Total Body Water

**Figure 6:**
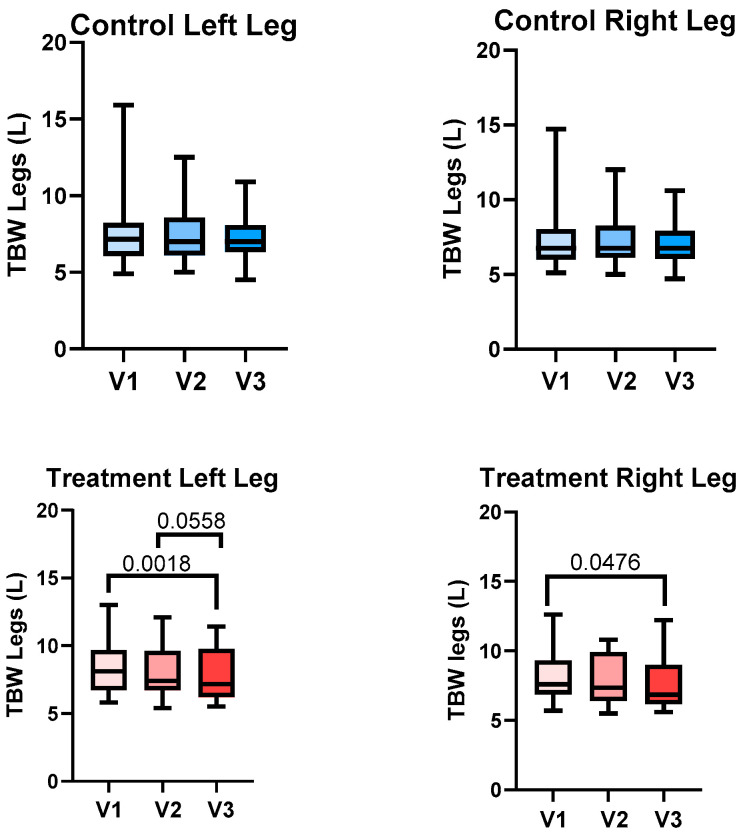
Total body water (mean ± SD) in the left and right legs of women with lipedema in the control (blue) and treatment (red) groups during Baseline Visit 1 (V1), pre-treatment with an advanced pneumatic compression device (APCD, Visit 2 (V2), and post-treatment Visit 3 (V3), assessed using bioimpedance spectroscopy (SOZO, Impedimed, Brisbane, Australia).

Combining all left legs (n = 46) and all right legs (n = 46) in this study, the mean total body water (TBW) of the left legs was 8.1 ± 2.2 L compared to the right, 7.8 ± 2.1 L (*p* = 0.0077). TBW significantly decreased in the right and left legs between V1 and V3 for the treatment group but not the control group (Figure 6), based on paired *t*-test results.

##### Total Body Water of the Legs

There was no significant difference in the TBW of the legs between V2 and V3 for the treatment group or control group for the right and left legs. Although not statistically significant, the reduction in TBW from V2 to V3 in the treatment group approached significance (*p* = 0.0558), suggesting a potential effect that may require further investigation with a larger sample size (6).

##### Extracellular and Intracellular Fluid

**Figure 7:**
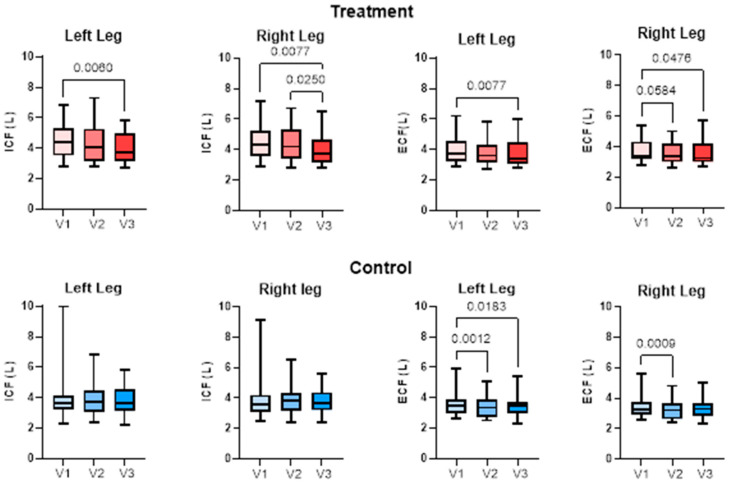
Intracellular fluid (ICF) and extracellular fluid (ECF) in the right and left legs of women with lipedema after advanced pneumatic compression device (APCD) use for 30 days (treatment) or no treatment (control). Fluid volumes (mean ± SD) were evaluated using bioimpedance spectroscopy for the right and left legs of the treatment (red) and control (blue) groups during Baseline Visit 1 (V1), pre-treatment Visit 2 (V2), and post-treatment (APCD Treatment group only) Visit 3 (V3).

Extracellular fluid (ECF) significantly decreased in the treatment group in the left leg from V1 to V3 and in the right leg from V1 to V3 and V2 to V3, assessed with paired t-test. Intracellular fluid (ICF) significantly decreased in the treatment group left leg from V1 to V3 and in the right leg from V1 to V3 and V2 to V3. ECF decreased in the left leg of the control group from V1 to V2 and V1 to V3 and in the right leg from V1 to V2 (Figure 7). There was no significant change in ICF in either leg of the control group.

#### 3.3.3. Caliper

There were no differences between caliper measurements on the right and left anterior and medial legs of women with lipedema in the treatment or control groups between visits (Table 2) assessed using paired t-tests. The caliper measurements of the abdomen significantly decreased for the treatment group and not the control group.

#### 3.3.4. Timed Up and Go

There was no difference in the Timed Up and Go test for the treatment group between V2 (12.32 ± 2.033 s) and V3 (12.73 ± 2.028 s) or the control group between V2 (11.75 ± 3.110 s) and V3 (11.58 ± 3.243 s) based on paired t-test results.

#### 3.3.5. Ultrasound Depth of SAT

##### Chronic Change in SAT Depth

**Figure 8:**
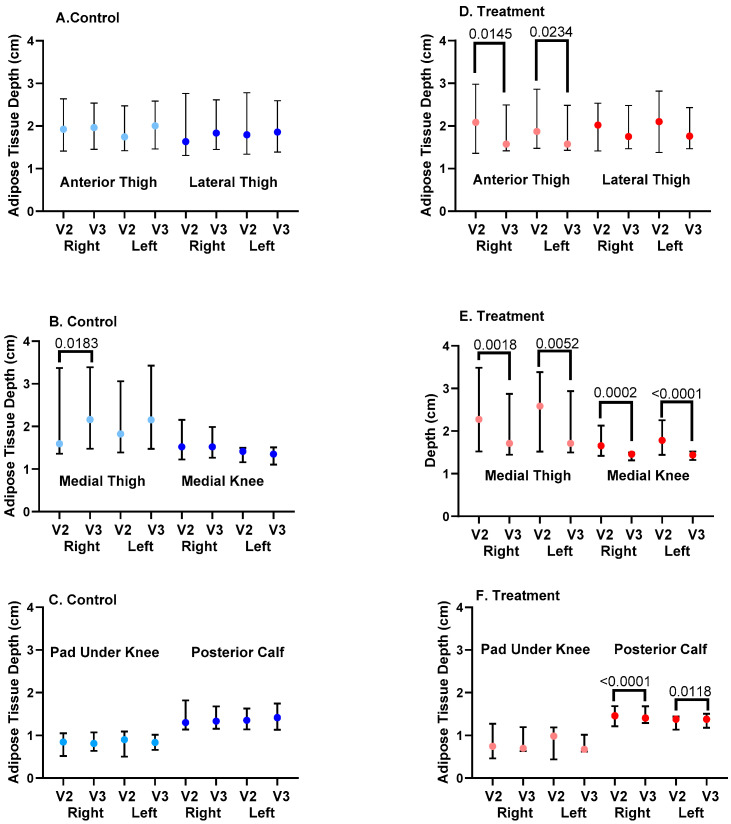
Subcutaneous adipose tissue depth after advanced pneumatic compression device (APCD) use in the treatment group or no treatment (control) group of women with lipedema. Depth measurements of adipose tissue in six locations on the right and left legs of women with lipedema were measured at Visit 2 (V2) and Visit 3 (V3) in the treatment (red) and control (blue) groups; V3 reflects changes after APCD use in the treatment group.

SAT depth significantly decreased from V2 to V3 for the treatment group at the right and left anterior thighs, medial thighs, medial knees, and posterior calves (Figure 8), assessed using paired t-tests. There was no significant change in SAT depth from V2 to V3 for control subjects at the anterior or lateral thigh, medial knee, or pad under the knee or posterior calf. SAT depth increased for the left and right medial thighs in the control group (Figure 8).

##### Acute Changes in SAT Depth

To assess if the APCD had acute effects, we examined SAT depth before and after one hour of APCD use at V2 and V3 and compared these values to the control group who received no intervention during the visits. There were no significant acute changes in SAT depth at six locations on the right and left legs for the control group (Table 3).

There was an acute and significant reduction in SAT depth for the anterior thigh on the right and left legs for the treatment group, with a sustained decrease during V3, so that acute changes were not found during V3 (Pre- and Post-APCD; Table 3; Figure 8). There were significant acute reductions in the size of the pad under the knee and the posterior calf of the right leg of the treatment group during V2, which were sustained for the posterior calf during V3. The medial thigh and medial knee pad of the right leg in the treatment group during V3 also decreased significantly and acutely (Table 3).

### 3.4. Qualitative Measures

#### 3.4.1. Visual Analog Scales

Symptoms of aching or throbbing, burning or stinging, tiredness or heaviness, swelling or tightness of the legs, difficulty walking, and easy bruising significantly reduced in the treatment group from V2 to V3. There were no significant changes in any of the leg symptoms in the control group from V2 to V3, except for swelling tightness of the legs (Table 4).

#### 3.4.2. RAND-SF-36

**Figure 9:**
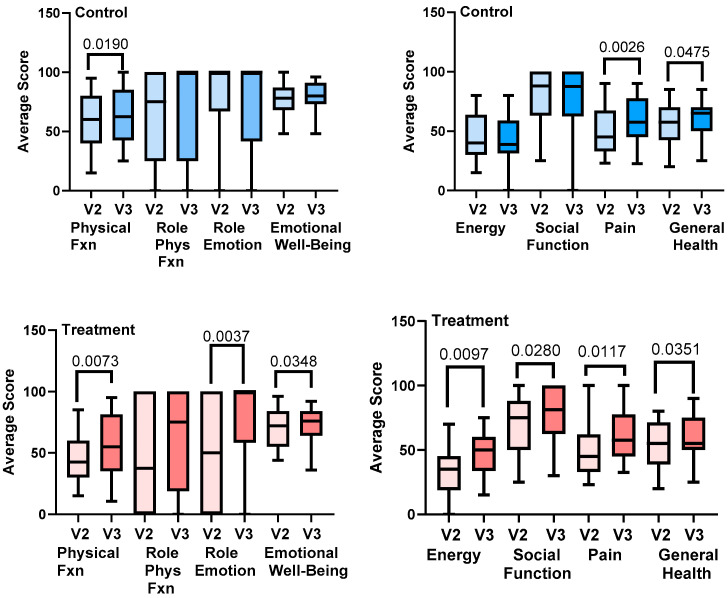
RAND SF-36 quality of life measurements after compression garment use alone (control) or combined with use of an advanced pneumatic compression device (APCD; treatment). The RAND SF-36 was administered during Visit 2 (V2) and Visit 3 (V3). Eight health concepts are shown for the control (blue) and treatment (red) groups: physical functioning, role limitations due to physical health problems, role limitations due to personal or emotional problems, emotional well-being, energy (vitality), social functioning, bodily pain, and general health perceptions. Fxn = function.

In the treatment group, there were significant improvements in physical function, emotional well-being and emotional role, energy, social function, pain, and general health from V2 to V3, assessed using paired t-tests. There was a significant improvement in physical function, pain, and general health between V2 and V3 in the control group (Figure 9).

#### 3.4.3. LEFS

There was a significant increase (improvement) in the value of the LEFS for the treatment group from V2 to V3 (61.3 ± 14.4 to 70.1 ± 16.2; *p* = 0.0009) but not in the control group (72.5 ± 14.6 to 72.9 ± 15.2; *p* = NS). There was a significant difference in the baseline LEFS scores for the control and treatment groups (*p* = 0.0129), with the treatment group starting at a lower level than the control group.

## 4. Discussion

This study aimed to evaluate the benefits of APCDs for women with lipedema, primarily Stage 2, without clinically evident lymphedema or active venous disease. There is controversy on whether there is edema in lipedema, with some authors supporting [2,34,35,36,37,38,39] and some against [40,41] this assertion. The data from this paper support the presence of edema in lipedema tissue, which is different from lymphedema in that it is not clinically apparent as pitting tissue or swelling of the feet or hands, yet it can be improved by an APCD.

To evaluate leg volume as one measure of leg edema, we utilized 3D imaging technology. There was no change in volume in the left or right leg for the control group and no change in volume in the right leg for the treatment group, yet there was a significant reduction in the mean volume of the left leg in the treatment group. In a study of 440 people with leg swelling, 52 of which had lipedema with lymphedema, the left leg was preferentially affected in unilateral and bilateral lymphedema [42]. The authors state that the left-sided lymphedema proclivity could be explained by chronic left iliac vein compression by the overlying right common iliac artery or, rarely, by the ipsilateral internal iliac artery (May–Thurner syndrome), accentuated by abdominal obesity, which is consistent with our data. Indeed, when combining all the left legs and all the right legs in this study, the mean TBW of the left legs was significantly higher when compared to the right legs. L-Dex measurements were also higher in the left legs than the right legs in both the treatment and control groups, indicating an increased risk of lymphedema due to increased fluid in the left legs over the right legs.

Tape measurements of the legs to calculate leg volume showed no change in this study for either the treatment or control groups. This may be due to different staff taking measurements during each visit. Even with rigorous training, user error contributes to a significant discrepancy in tape measurement data for leg volume. In addition, measuring leg circumference in serial measurements using a tape measure is tedious and labor-intensive [43]. In this study, tape measurements were made every 10 cm from the ankle to the thigh; measurements every 4 cm may have improved accuracy but could have also contributed further to error.

To more accurately measure fluid changes in the legs, we employed the use of BIS to measure the TBW of the legs and risk of lymphedema. The use of an APCD significantly reduced TBW in the legs in the treatment group and not the control group; in fact, there was an increase in volume in the left legs of controls. The APCD also reduced the risk of lymphedema in legs in the treatment group and not the control group based on L-Dex measurement, which compares fluid in the legs to fluid in the arms, where arms tend to be less affected than legs in lipedema. L-Dex correlates with limb volume based on perometry, degree of pitting edema, lymphoscintigraphy, and lymphedema staging using indocyanine green (ICG) lymphography [44]; therefore, the L-Dex measurement provides a strong link between lipedema and lymphedema in this study.

We also investigated whether there was a change in the location of fluid in the leg, specifically in the intracellular and extracellular spaces. The extracellular space includes the interstitial space between cells and outside vascular walls, as well as plasma inside blood vessels [45]. About 60–80% by weight of adipose tissue is lipid, 5–30% is water, and the remaining 2–3% is protein [46]. The fluid in the interstitial space is free-flowing, but there are also proteoglycan filaments which bind water and salt, forming a “tissue gel” [2] or ground substance. Extracellular fluid in the legs decreased after compression garment use in the control group, confirming that compression wear is important in the treatment of lipedema. The fluid within cells, the ICF, did not change in the control group. With APCD use, there was a decrease in both ICF and ECF. When body water loss is minimal, the water deficit comes primarily from the extracellular space; as more body water is lost, a proportionately greater percentage of the water deficit comes from the intracellular space [47,48], consistent with the reduction in ECF by compression garments alone and reduction in ECF + ICF by the addition of the APCD.

The ground substance accounts for only a small percentage of the volume fraction in adipose tissue, and free fluid is negligible [49]. On subjecting adipose tissue to a uniaxial compressive strain of 50%, the volume of liquid expressed from adipose tissue was less than 1% of the overall volume [50]. However, the interstitial space is larger in lipedema tissue [51,52], tissue salt, which binds proteoglycans, is higher in adipose and muscle tissue in the legs of women with lipedema [53], and lymphatic vessels pump at higher rates in women with early lipedema [11], suggestive of increased interstitial fluid. Future research could include assessments of compressive strain studies to better understand fluid in lipedema tissue.

For our study, the question becomes which cells reduced their intracellular fluid? Cells maintain their shape and water content owing to the difference in solute concentration, where the cytoplasm of a cell has higher osmotic and hydraulic pressure than the immediate surroundings [54]. Cells are also sensitive to changes in hydraulic and osmotic pressure around them. For example, when hydrostatic pressure was increased by 70 mm Hg around leukocytes (immune cells) for 2 h, the radius of the cells and intracellular sodium and potassium ion concentrations decreased, causing cell shrinkage [54]. Ion movement should induce osmosis-driven water flow across the membrane, which is likely the reason for the observed volumetric deformation of the immune cells. Since water can move freely across the cell membrane between the intracellular and interstitial space, the APCD in our study likely changed the osmotic gradient, which is required for fluid movement between cells in the leg tissue [55]. Changing the osmotic gradient across cell membranes may allow for longer periods of time of fluid reduction after treatment with an APCD versus compression alone. Future studies should consider long-term evaluations of fluid reduction after APCD use in women with lipedema.

The leg grossly contains skin, skeletal muscles, connective tissue (including bone, tendons and ligaments), nerves, blood and lymphatic vessels, and cells including adipocytes, fibroblasts, myocytes, immune cells, or other cells. Each adipocyte comprises a single lipid vacuole and a nucleus within a phospholipid bilayer, in a collagen mesh. Due to the high intracellular lipid droplet content, adipose tissue functions as an incompressible inviscid fluid [50]. Skeletal muscle on the other hand comprises 75% water [47,48]. Women with lipedema have poor muscle function in their legs [56], but no ultrastructural studies on the muscles of women with lipedema are available. The reduction in ICF in the legs of women with lipedema may therefore have resulted from a reduction in water from adipocytes, myocytes, and/or immune cells, amongst others. A focus on muscle architecture in treatment studies may provide additional insight into the pathophysiology of lipedema.

The APCD may have also reduced intravascular fluid as well. This is consistent with the APCD pump significantly reducing leg volume, observed by 3D imaging, which thereby reshaped the limbs. Reducing excess fluid in the extracellular space is important for reducing the feeling of heaviness in the legs, but a reduction in fluid in the vessels may reduce fluid available to leak through abnormally permeable endothelial cells into lipedema tissue [8,9,10]. These data highly suggest that there is edema in the tissue of women with lipedema and this edema can be effectively reduced in multiple compartments using an APCD. In addition, use of an APCD might reduce the risk of developing lymphedema in women with lipedema.

Lipedema is primarily considered to be a disease of subcutaneous adipose connective tissue. In addition to fluid reduction, we wanted to determine if an APCD could reduce lipedema SAT depth. Use of the APCD was associated with reductions in SAT depth near the umbilicus, based on caliper measurements, and SAT depth on the anterior and medial thigh, medial knee, and posterior calves, based on ultrasound. There were also significant reductions in some areas of the lipedema SAT right after use of the APCD during a single study visit, suggesting an acute reduction in a component of the SAT rather than long-term structural changes (see below). These changes were not found in the control group, suggesting that reductions in SAT depths were due to the APCD. In the women who did not use an APCD (Controls), there was an increase in medial thigh SAT depth. The abdomen SAT reduction based on caliper measurements supports the use of APCD garments that treat the torso along with the legs in women with lipedema who have affected abdomens along with legs.

It is unclear if the reduction in SAT depth after use of an APCD is a direct reduction in adipose tissue or other components of the tissue (such as fibrosis) or simply reflects the decrease in tissue fluid after the use of the APCD. Under a hypertonic pressure of 400 mosmol, half of adipocytes lose visible lipid droplets, and the remaining adipocytes also experience a decrease in lipid droplet size and adopt a spindle-like shape consistent with de-differentiation into mesenchymal stem cells [57]. The reduction in SAT depth could therefore reflect a change in the adipocytes themselves.

Lipedema tissue is painful, and it has been proposed that this is neuropathic pain due to inflammation [58]. Use of the APCD reduced many symptoms of pain in the legs, whereas a similar reduction was not found in the control group, suggesting an APCD can improve symptoms of lipedema associated with pain over that of compression alone. While there was a reduction in body pain on the RAND SF-36 in both the treatment and control groups, the VAS specifically targeted tenderness (pain) in the legs and therefore is a better representation of lipedema pain in this study, where lipedema pain was reduced by the use of an APCD.

A reduction in fluid and SAT in women with lipedema are important, but it needs to translate to an improvement in quality of life and function. There was no change in the TUG functional test for the women in this study. It should be noted, however, that the time it took for women to complete the test was close to the mean for 80–84-year-old women in the Norwegian Tromsø Study and similar to a meta-analysis of TUG data [59]. These data suggest that women with lipedema with average age of 50 years are likely functionally impaired by their lipedema. It may require more than 3 months of APCD use to translate into improved function.

While there were no significant changes in objective functional measures in this study after APCD treatment, there was a significant improvement in quality-of-life measures using the RAND SF-36 in the control group, specifically physical function, pain, and general health, likely reflecting improvements due to compression garments, and in the treatment group in physical function, emotional well-being, role of emotion, energy, social function, pain, and general health, reflecting improvement due to the use of compression garments and the APCD. Women’s social and emotional function improving after use of an APCD suggests improvement in mental health in addition to physical health, which was not seen with compression garments alone. In addition, there was a significant improvement in the LEFS, with questions about daily life tasks and leg symptoms, in the treatment group but not the control group. The APCD therefore significantly improved quality of life women living with lipedema over that of compression garments alone. There was a significant difference in the starting value of the LEFS at V2 between the treatment and control groups, but this is likely due to variance within the population.

The WHR of the women in this study was below the threshold set for central obesity in women of greater than 0.8 [60], despite the mean BMI exceeding criteria for obesity (>30 kg/m^2^). The mean WHtR was just under the cutoff of 0.6 for a reasonable future risk of death and cardiovascular events set for women aged 50 years or older in a study of 5956 women from Germany [61]. Other estimates state a WHtR 0.5–0.59 indicates increased health risks and that people with a WHtR of 0.6 or more have further increased health risks [62].

The study population in this paper had high levels of anxiety, which is known to be a co-morbidity in the hypermobile joint syndrome population [63]. Also notable in this study population were high levels of allergies reaching almost 100% of participants, in agreement with published data [12]. Mast cell activation syndrome is linked to allergies [64] and may be linked to lipedema, with investigators finding high levels of histamine and its metabolites in tissue samples from women with lipedema compared to controls; histamine levels reduced after treatment with sodium cromoglycate, suggesting a reduction in mast cell activity [65]. These data suggest that lipedema is more likely than not a systemic disease rather than a focal disease of the arms and legs.

## 5. Conclusions

The APCD used in this study for 30 days by women with lipedema who also wore 20–30 mm Hg compression leggings daily significantly reduced tissue fluid and SAT depth and improved quality of life, pain, and leg symptoms over women who wore compression garments alone. Advanced pneumatic compression devices should be considered for the conservative care of women with lipedema who present with symptoms of leg heaviness, swelling, and pain. Finally, this study lends support to the presence of edema in lipedema.

## Figures and Tables

**Table 1:** Demographics of the study group.

Demographic	Group (N)		*p*-Value *	Total
	Control (24)	Treat (22)		
Age	48.8 ± 8.5	52 ± 9.2	NS	50.3 ± 8.9
Female/Male	24/0	22/0	NS	46
Race: White/Black	23/2	16/5	NS	39/7
Systolic BP (mmHg)	122.5 ± 16	124.7 ± 14	NS	123.5 ± 15
Diastolic BP (mmHg)	79.3 ± 8.5	80.5 ± 8	NS	72.8 ± 9
Heart rate (bpm)	73.5 ± 8.3	72 ± 10.4	NS	72.8 ± 9
Height (cm)	161 ± 7	161.6 ± 6.6	NS	161.3 ± 6.7
Weight (kg)	94.9 ± 23	104.7 ± 22	NS	99.4 ± 22.7
Waist (cm)	92.4 ± 13	98.4 ± 11.4	NS	95.2 ± 13
Hips (in)	121.1 ± 17	130.4 ± 15	NS	125.3 ± 6.7
WHR	0.76 ± 0.04	0.75 ± 0.05	NS	0.76 ± 0.05
WHtR	0.57 ± 0.08	0.61 ± 0.07	NS	0.59 ± 0.07
BMI (kg/m^2^)	35.3 ± 7	38.9 ± 7.5	NS	37 ± 7.4
**Lipedema Stages**	**Control (number)**		**Treatment (number)**	
1	0		0	
2	23		20	
3	1		2	
**Medical History**	**Control (%)**		**Treatment (%)**	
Migraine +/− aura	20/24		33/14	
Depression	24		48	
Anxiety	40		52	
Allergies	100		95	
Hypertension	8		29	
Prediabetes/Diabetes	20/12		14/9.5	
Pregnant (ever)	84		86	
Miscarriage	32		33.3	
Birth Control	92		90	
Menopause	40		62	
Asthma COPD	16		28.5	
Thyroid Disease	28		38	
	**Control (Mean ± SD)**		**Treatment (Mean ± SD)**	
Age 1st menstruation	12.4 ± 1.9		12.2 ± 1.3	
Times pregnant	2.3 ± 1.7		2.1 ± 2.0	
Age menopause	50.2 ± 4.2		46.7 ± 7	
Surgical History	**Control (%)**		**Treatment (%)**	
Bariatric	16		33.3	
Gastrointestinal	64		52	
Musculoskeletal	32		43	
Reproductive	72		67	

* NS = non-significant; BMI = body mass index; BP = blood pressure; COPD = chronic obstructive pulmonary disease; WHR = waist-to-hip ratio; WHtR = waist-to-height ratio.

**Table 2:** Caliper measurements (mean ± SD; mm) at locations of lipedema tissue in control and treatment groups during Visit 2 (before advanced pneumatic compression device [APCD] treatment) and Visit 3 (after APCD use for 30 days treatment group only).

Location	Visit 2	Visit 3	*p*-Value
**Control Group**			
Under Umbilicus	40.3 ± 17.3	40.5 ± 14.8	NS
Right Anterior Thigh	61.1 ± 6.3	62.2 ± 4.8	NS
Right Medial Thigh	55.2 ± 9.1	56.9 ± 7	NS
Left Anterior Thigh	60.8 ± 6.4	62.6 ± 5	NS
Left Medial Thigh	55.5 ± 9.3	57.9 ± 7	NS
**Treatment Group**			
Under Umbilicus	45.1 ± 14.3	38.9 ± 14.4	0.0279
Right Anterior Thigh	63.8 ± 4.7	64.1 ± 4.4	NS
Right Medial Thigh	57.1 ± 8	58.8 ± 7	NS
Left Anterior Thigh	62.2 ± 6.8	64.1 ± 4	NS
Left Medial Thigh	58.1 ± 7.5	59 ± 9	NS

**Table 3:** Adipose tissue depth (cm) at six locations on the legs of women with lipedema during Visit 2 before any treatment except compression garment use in all women and Visit 3 after 30 days of treatment with an intermittent pneumatic compression device (APCD). During each visit, adipose tissue depth was measured before (pre) and after (post) one hour of APCD treatment in the treatment group or without intervention in the control group.

**Adipose Tissue Location**	**Control (n = 24)**					
	**Visit 2**		***p*-Value**	**Visit 3**		***p*-Value**
	**Pre**	**Post**		**Pre**	**Post**	
**Right Leg**						
Anterior thigh	2.2 ± 1	2.1 ± 1.0	NS	2.3 ± 1.0	2.3 ± 1.0	NS
Lateral thigh	2.1 ± 1.1	2.1 ± 1.0	NS	2.2 ± 0.9	2.2 ± 0.9	NS
Medial thigh	2.3 ± 1.2	2.3 ± 1.3	NS	2.5 ± 1.1	2.5 ± 1.2	NS
Medial knee	1.9 ± 1.1	1.6 ± 0.7	NS	1.5 ± 0.5	1.4 ± 0.5	NS
Pad under knee	0.84 ± 0.4	0.8 ± 0.4	NS	0.94 ± 0.5	0.94 ± 0.5	NS
Posterior calf	1.44 ± 0.5	1.4 ± 0.4	NS	1.4 ± 0.4	1.4 ± 0.4	NS
**Left Leg**						
Anterior thigh	2.1 ± 0.9	2.2 ± 0.9	NS	2.2 ± 1.0	2.2 ± 1.1	NS
Lateral thigh	2.1 ± 1.0	2.1 ± 0.8	NS	2.2 ± 0.9	2.2 ± 0.9	NS
Medial thigh	2.3 ± 1.2	2.3 ± 1.2	NS	2.5 ± 1.1	2.5 ± 1.2	NS
Medial knee	1.7 ± 0.7	1.6 ± 0.7	NS	1.4 ± 0.5	1.4 ± 0.5	NS
Pad under knee	0.82 ± 0.4	0.89 ± 0.4	NS	1.0 ± 0.5	0.95 ± 0.5	NS
Posterior calf	1.5 ± 0.5	1.4 ± 0.4	NS	1.4 ± 0.4	1.5 ± 0.4	NS
**Adipose Tissue Location**	**Treatment (n = 22)**					
	**Visit 2**		***p*-Value**	**Visit 3**		***p*-Value**
	**Pre**	**Post**		**Pre**	**Post**	
**Right Leg**						
Anterior thigh	2.3 ± 1.0	2.1 ± 0.9	0.009	2.1 ± 0.8	2.0 ± 0.8	NS
Lateral thigh	2.2 ± 0.9	2.1 ± 0.8	NS	2.1 ± 0.6	2.0 ± 0.6	NS
Medial thigh	2.6 ± 1.2	2.5 ± 0.9	NS	2.3 ± 1.0	2.2 ± 0.9	0.0097
Medial knee	1.9 ± 0.7	1.7 ± 0.5	NS	1.5 ± 0.3	1.4 ± 0.2	0.0097
Pad under knee	0.86 ± 0.4	0.74 ± 0.4	0.025	0.9 ± 0.4	0.9 ± 0.4	NS
Posterior calf	1.5 ± 0.3	1.4 ± 0.3	0.036	1.4 ± 0.2	1.3 ± 0.2	NS
**Left Leg**						
Anterior thigh	2.2 ± 1.0	2.1 ± 0.9	0.007	2.1 ± 0.8	2.0 ± 0.8	NS
Lateral thigh	2.2 ± 0.8	2.0 ± 0.7	NS	2.0 ± 0.6	1.9 ± 0.6	NS
Medial thigh	2.6 ± 1.2	2.3 ± 1.0	NS	2.3 ± 1.0	2.2 ± 0.9	NS
Medial knee	1.9 ± 0.6	1.8 ± 0.5	NS	1.5 ± 0.2	1.5 ± 0.3	NS
Pad under knee	0.86 ± 0.4	0.8 ± 0.4	NS	1.0 ± 0.7	0.8 ± 0.3	NS
Posterior calf	1.5 ± 0.3	1.4 ± 0.3	NS	1.4 ± 0.2	1.4 ± 0.5	NS

**Table 4:** Subject visual analog scale ratings of signs and symptoms for the right and left leg during Visit 2 before any treatment except compression garment use in all subjects, and during Visit 3 after treatment with an intermittent pneumatic compression device (APCD) for 30 days in the treatment group only.

Location	Control (n = 24)			Treatment (n = 22)		
	Visit 2	Visit 3	*p*-Value	Visit 2	Visit 3	*p*-Value
**Right Leg**						
Tender	4.7 ± 2.2	3.7 ± 1.9	NS	4.6 ± 2.2	2.5 ± 1.7	0.0003
Ache throb	4.1 ± 2.3	2.9 ± 2.4	NS	4.5 ± 2.4	1.3 ± 1.8	<0.0001
Burn Sting	2.2 ± 2.4	1.7 ± 1.9	NS	2.6 ± 2.4	0.59 ± 1.3	0.0016
Tired Heavy	5.0 ± 2.3	4.0 ± 2.5	NS	5.5 ± 2.5	1.9 ± 1.9	<0.0001
Swelling Tightness	4.8 ± 2.5	3.7 ± 2.4	0.0377	4.5 ± 2.6	1.5 ± 1.5	0.0005
Difficulty Walking	2.5 ± 2.9	1.8 ± 2.0	NS	4.1 ± 2.8	0.7 ± 1.3	<0.0001
Easy bruising	5.9 ± 2.89	5.0 ± 2.5	NS	5.9 ± 3.0	3.3 ± 3.2	0.0016
**Left Leg**						
Tender	4.7 ± 2.2	3.7 ± 1.9	NS	5.0 ± 2.0	2.5 ± 1.7	<0.0001
Ache Throb	4.1 ± 2.3	2.9 ± 2.4	NS	4.8 ± 2.2	1.3 ± 1.8	<0.0001
Burn Sting	2.2 ± 2.4	1.7 ± 1.9	NS	2.6 ± 2.4	0.59 ± 1.3	0.0008
Tired Heavy	5.0 ± 2.3	4.0 ± 2.5	NS	5.6 ± 2.4	1.9 ± 1.9	<0.0001
Swelling Tightness	4.8 ± 2.5	3.7 ± 2.4	NS	5.1 ± 2.6	1.4 ± 1.6	<0.0001
Difficulty Walking	2.5 ± 2.9	1.8 ± 2.0	NS	4.2 ± 2.8	0.73 ± 1.3	<0.0001
Easy Bruising	5.9 ± 2.9	5.0 ± 2.5	NS	5.9 ± 3.0	3.3 ± 3.2	0.0011

## Data Availability

The data presented in this study are available on request from the corresponding author due to proprietary nature of the information as it pertains to Lympha Press.

## Abbreviations

The following abbreviations are used in this manuscript:

APCD	Intermittent pneumatic compression device
BIS	Bioimpedance spectroscopy
BMI	Body mass index
COPD	Chronic obstructive pulmonary disease
CVD	Cardiovascular disease
CVI	Chronic venous insufficiency
ECF	Extracellular fluid
ICF	Intracellular fluid
ICG	Indocyanine green
HTN	Hypertension
MCAS	Mast cell activation syndrome
NS	Non-significant
PCOS	Polycystic ovarian syndrome
POTS	Postural orthostatic tachycardia syndrome
SAT	Subcutaneous adipose tissue
SF-36	RAND Short Form 36-Item Survey
US	Ultrasound
VAS	Visual analog scales
WHR	Waist-to-hip ratio
WHtR	Waist-to-height ratio
